# Reanalysis of putative ovarian follicles suggests that Early Cretaceous birds were feeding not breeding

**DOI:** 10.1038/s41598-020-76078-2

**Published:** 2020-11-04

**Authors:** Gerald Mayr, Thomas G. Kaye, Michael Pittman, Evan T. Saitta, Christian Pott

**Affiliations:** 1 Ornithological Section, Senckenberg Research Institute and Natural History Museum Frankfurt, Senckenberganlage 25, 60325 Frankfurt am Main, Germany; 2 Foundation for Scientific Advancement, 7023 Alhambra Drive, Sierra Vista, Arizona 85650 USA; 3 Vertebrate Palaeontology Laboratory, Division of Earth and Planetary Science, The University of Hong Kong, Pokfulam, Hong Kong SAR China; 4 Integrative Research Center, Life Sciences Section, Field Museum of Natural History, 1400 South Lake Shore Drive, Chicago, IL 60605 USA; 5 LWL-Museum of Natural History, Westphalian State Museum with Planetarium, Sentruper Straße 285, 48161 Münster, Germany

**Keywords:** Palaeontology, Palaeoecology

## Abstract

We address the identity of putative ovarian follicles in Early Cretaceous bird fossils from the Jehol Biota (China), whose identification has previously been challenged. For the first time, we present a link to the botanical fossil record, showing that the “follicles” of some enantiornithine fossils resemble plant propagules from the Jehol Biota, which belong to *Carpolithes multiseminalis*. The botanical affinities of this “form-taxon” are currently unresolved, but we note that *C. multiseminalis* propagules resemble propagules associated with cone-like organs described as *Strobilites taxusoides*, which in turn are possibly associated with sterile foliage allocated to *Liaoningcladus.* Laser-Stimulated Fluorescence imaging furthermore reveals different intensities of fluorescence of “follicles” associated with a skeleton of the confuciusornithid *Eoconfuciusornis zhengi*, with a non-fluorescent circular micro-pattern indicating carbonaceous (or originally carbonaceous) matter. This is inconsistent with the interpretation of these structures as ovarian follicles. We therefore reaffirm that the “follicles” represent ingested food items, and even though the exact nature of the *Eoconfuciusornis* stomach contents remains elusive, at least some enantiornithines ingested plant propagules.

## Introduction

Over the past decades, the Jehol Biota in northeast China yielded an extraordinary diversity of fossils, which produced unprecedented insights into Early Cretaceous ecosystems. Even though the specimens from these localities are known for their exquisite soft-tissue preservation, the discovery of putative ovarian follicles in some of the bird fossils stands out and is otherwise unmatched in the avian fossil record.

Aggregations of such ovoid structures were first reported in specimens of the Enantiornithes and Jeholornithidae^1^. Their identification as mature ovarian follicles was, however, challenged^2^, because (1) such perishable structures are unlikely to be preserved in multiple fossils that otherwise show little or no preservation of comparable soft tissue types and would have to have survived through decay and diagenesis; (2) the putative “follicles” have similar dimensions in very differently-sized birds (Table 1); and (3) based on evidence from paired eggs in situ, non-avian maniraptorans already had the ovulation mode of extant birds, that is, a consecutive maturing of follicles, so that a simultaneous maturing of multiple follicles in early diverging birds would be unexpected^2^. The first two of these observations were countered by a reference to eggs in Jehol fish fossils that remain unpublished and by the proposal that a similar follicle size may have been due to similar dimensions of the pelvic canal in differently-sized Mesozoic birds^3^. Here it is noted, however, that it is not the mere preservation of ovarian follicles as such that was deemed unlikely, but their occurrence in fossils that do not show other traces of preservation of comparable soft tissue types^2^. The third argument, that is, the presence of an avian-like ovulation mode in non-avian theropods (which conflicts with the presence of multiple mature ovarian follicles), was not addressed. This point was subsequently reaffirmed by the notion that a simultaneous maturing of ovarian follicles is unlikely to occur in volant animals^4^.

**Table 1:** Overview of birds from the Jehol Formation with “follicle”-like plant propagules preserved as stomach contents.

Taxonomic identification	Specimen number	Propagule size	Femur length
*Jeholornis prima* (Jeholornithidae)	STM 2-51	7.1‒8.8 mm^1^	108.8 mm^6^
*Eoconfuciusornis zhengi* (Confuciusornithidae)	STM 7-144	3.5‒5.8 mm^8^	25.4 mm^8^
Enantiornithes indet.	STM 29-8	5.8‒8.8 mm^1^	40.2 mm^6^
Enantiornithes indet.	STM 10-45	6.7‒8.8 mm^1^	34.9 mm^6^
Enantiornithes indet.	STM 10-4	7.2 mm^6^ (mean value)	38.7 mm^6^
Enantiornithes indet.	STM 10-12	7.7 mm^6^ (mean value)	‒
Enantiornithes indet.	STM 11-121	6.8 mm^6^ (mean value)	37.5 mm^6^
Enantiornithes indet.	STM 11-212	5.4 mm^6^ (mean value)	~ 32‒35^6^
*Linyiornis amoena* (Enantiornithes)	STM 11-80	5.6‒7.1^7^ mm	37.7 mm^6^

To meet the increased calcium demands of eggshell formation, female birds develop a special tissue in the medullary cavity of their bones prior to and during oviposition. Such medullary bone has not been identified in specimens with presumed “follicles”^5^, and the histological data therefore do not lend support to the identification of these structures as mature ovarian follicles.

**Figure 1:**
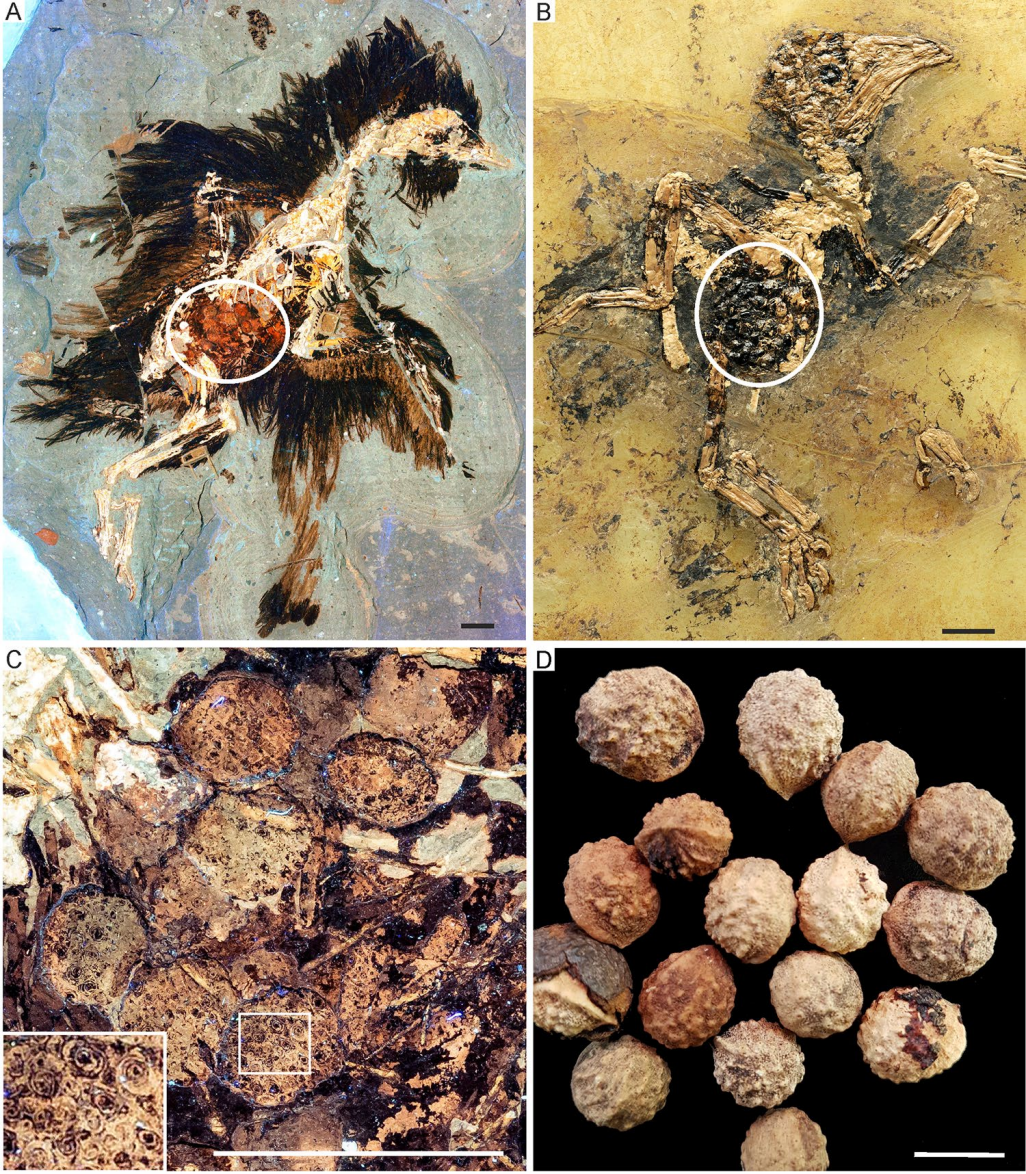
Stomach contents of *Eoconfuciusornis zhengi* (Confuciusornithidae) in comparison to those of an Eocene bird and extant plant propagules with a similar morphology. (**A**) LSF-image of the specimen of *Eoconfuciusornis zhengi* (STM 7-144) from the Early Cretaceous Huajiying Formation with “follicle”-like structures (encircled). (**B**) Skeleton of the stem group mousebird *Selmes absurdipes* (Coliiformes) from the late early/early middle Eocene of Messel in Germany (SMF-ME 2375) with angiosperm seeds preserved as stomach contents (encircled). (**C**) Detail of the supposed follicles of *E. zhengi* under LSF; the inset in the bottom left shows a magnified detail of the circular/spiralling micropattern of the “follicles” in the framed area; concerning equalization, saturation and color balance, the image was post processed with Photoshop CS6 (adobe.com). (**D**) Ovules (fleshy epimatium removed) of extant *Afrocarpus falcatus* (Podocarpaceae; from https://en.wikipedia.org/wiki/Afrocarpus_falcatus, modified; the original photo was published under a CC-BY-SA-4.0 license by Paul Venter). Scale bars equal 10 mm.

Irrespective of these objections, however, further examples of putative fossilized “follicles” were described in fossils of the Enantiornithes^6,7^ and the confuciusornithid *Eoconfuciusornis zhengi*^8^ (Fig. 1A). Identification of ovarian follicles formed the basis for hypotheses on the paleobiology of early birds^9,10^, and these structures have now been reported for *Jeholornis* (Jeholornithidae), *Eoconfuciusornis* (Confuciusornithidae), and at least seven enantiornithine specimens^1,6–8^. The presence of similar structures was furthermore noted in a fossil of the non-avian coelurosaurian theropod *Compsognathus*^2^, in which they were also interpreted as ovarian follicles^3,6^ rather than being of taphonomic or diagenetic origin as previously assumed^11^.

The presumed ovarian follicles of Early Cretaceous birds have varying morphologies in different individuals. Whereas those initially reported^1^ are flat structures with a featureless or reticulate surface, those of *Eoconfuciusornis* exhibit a surface with concentric or spiraling circles^8^. Fossilization of ovarian follicles as such would be quite unexpected in fossils that otherwise show little evidence for preservation of similar soft tissue types, but their different surface textures in multiple specimens cast further doubts on their identification.

Most recently, the histology of these structures was studied and considered to be in concordance with their presumed identity as ovarian follicles^12^. However, as detailed below, we find several shortcomings in the evidence used to support these interpretations, which likely undermine an identity as ovarian follicles.

It therefore seems appropriate to comment again on the identity of the Jehol “follicles”. In the present study, we identify plant propagules (ovules, seeds, fruits, or parts thereof) in the botanical record of the Jehol Biota that are similar to some of the presumed “follicles” and comment on the possible phylogenetic affinities of these propagules. Moreover, we analyze the presumed “follicles” of *Eoconfuciusornis zhengi* with Laser-Stimulated Fluorescence imaging (LSF), which yields new insights into the texture and possible composition of these structures.

## Results

### Reexamination of the histological evidence for putative ovarian follicles

Bailleul et al.^12^ propose that microscopy, histochemical staining, and energy-dispersive spectroscopy reveal smooth muscle, collagen fiber, and blood vessel preservation within a putative perifollicular membrane of enantiornithine specimen STM 10-12. The authors acknowledge that the purported follicles vary in texture and morphology across nine referenced specimens and ‒ unlike the heterogeneity seen in modern bird follicle development ‒ are homogenous in size within each specimen. They attribute such peculiarities to evolutionary trends rather than lines of evidence against an ovarian identity.

We do not see clearly portrayed and unambiguously identified collagen and muscle fibers, vessels, sub-endothelial connective tissue, or intravascular material in the microscopy images presented^12^. Microscopic structural data is at risk of subjective interpretation^13^, and such considerations have often been invoked with respect to controversial Archaean ‘microfossils’^14^ and dubious dinosaur erythrocytes^15^. Concerning the Jehol fossils, features that were identified as purported blood vessels^12^ show little signs of the branching typical of well-preserved vessels, which the authors^12^ acknowledge. An expectation that the structures are preserved smooth muscle from follicles might also explain why they were described as pale pink^12^ rather than, more appropriately, brown. Sample manipulation by Bailleul et al.^12^ may also have added further noise to structural data that is already difficult to interpret—namely their coined process of ‘artificial decompaction’ during paraffin histological analysis.

When applied to geologic samples, certain biological methods are at risk of yielding false and misleading results. For example, recent authors^16^ failed to detect a proteinaceous signature in fossil feathers that had previously tested positive with antibodies raised against keratin^17^. The most likely source of this suspected false positive was attributed to adsorption of antibodies to the glue from excavation and preparation that was found coating the fossil feather specimen. Thus, the thick layer of glue or other organic consolidant on and infiltrating the purported follicle of STM 10-12 is concerning.

Infiltrating organic consolidants may have produced the structures reported as soft tissues in the demineralized paraffin sections^12^ and might also have influenced their subsequent histochemical staining. Masson’s trichrome staining uses a series of dyes designed to sequentially stain tissue components (e.g., muscle and collagen) known to be present within modern biological samples in different colors, influenced in part by the differing permeability of these components^18,19^. For the Jehol fossils, it was implicitly assumed that Masson’s trichrome stains these components to the exclusion of other substances with specificity^40^. However, this is not necessarily the case^19,20^. Variable permeability could arise from infiltrating consolidants. In their demineralized section, the predominant substance, possibly consolidant, is stained red from the earlier dye, while the later green stain simply localizes in the spaces between. This raises concern about how the section was interpreted when using a histochemical methodology not designed for analyzing fossils.

The layer identified as preserved follicle in the non-demineralized cross section is also peculiar because it is birefringent. This is more consistent with mineral rather than organic material, so much the more as no visible cells were reported in the non-demineralized cross section^12^. The layer appears to contain aluminosilicate clay mineral(s) because under energy-dispersive spectroscopy it is depleted in C relative to the consolidant, but is enriched in Al, Si, and O. Bailleul et al.^12^ distinguish this purported soft tissue layer from what they describe as a layer of organic material in the sediment below it. EDTA (ethylenediaminetetraacetic acid) is commonly used to demineralize bone apatite through chelation with Ca ions^21^. The possibility that other inorganic minerals in their sample might better persist to some degree through the implemented EDTA demineralization and subsequently affect the Masson’s trichrome staining should be tested with further experiments. However, this point is moot because the inorganic signature of the purported follicle layer contradicts the histochemical methodology used by Bailleul et al.^12^, which was interpreted under the assumption that the stained materials are organic collagen and muscle.

Although Bailleul et al.^12^ posit that fossil plant tissue would show diagnostic cellular structures, it is also possible to have organic preservation of a more amorphous kerogen derived from plant tissues^22,23^. Regardless, a plant origin of the spherical structures in the body cavity might still be consistent with the inorganic signatures detected in STM 10-12. Plant fossils have been known to pyritize^24^, which results in a loss of cellular structure preservation and occurs alongside rapid, early-stage organic tissue loss^25^. Pyritization has also been reported specifically in Jehol Group plant seeds^26^. Pyrite can be subsequently oxidized^27^, consistent with the reported Fe and O but lack of S, and this iron oxide pseudomorph of pyrite has been observed on Jehol Group insects^28^. Although plant tissues, including seeds, have in some rare instances been reported as phosphatized remains^29–31^, the limited Ca detected in STM 10-12 suggests that this is not the case here. While phytoliths are sometimes rare in seeds^32^, their presence could contribute in part to the prominent Si detected; even though Bailleul et al.^12^ report that no phytoliths were found, their methods and data in relation to this are not presented. Bailleul et al.^12^ suggest a process similar to clay mineral templating^33,34^ occurring based on the elemental signatures, but the preservation of plants in such a manner does not appear to be commonly reported in the literature as far as we can determine, and such clay templating occurs during late-stage metamorphism^35^ that is not associated with Jehol fossils.

The taphonomic models used by Bailleul et al.^12^ to support their follicle hypothesis (e.g., Ref. 36) were accepted uncritically, even though there is not a consensus within the discipline^37^. Their model of tissue preservation involves either (1) authigenic aluminosilicate replacement or (2) stabilization by environmental or hemoglobin-derived iron. These two proposed mechanisms would presumably yield either inorganic or organic preservation of the soft tissue, respectively, with implications for the validity of the histochemical staining. However, as discussed above, it is unclear whether or not Bailleul et al.^12^ argue for organic preservation of the soft tissues. More problematic is the preservation pattern within the specimens. Ovarian tissue would be expected to decay rapidly through microbial or autolytic action, but even if we allow for a scenario in which a carcass is rapidly deposited in an anoxic decay-limited lakebed, then their model would predict extensive soft tissue preservation in addition to follicles. The lack of other muscles (e.g., smooth muscle of the gastrointestinal tract), connective tissues, and blood vessels in these specimens is inconsistent with a taphonomic environment conducive to the preservation of these organic materials. Instead, the observed pattern is more consistent with these structures representing a fundamentally different type of biological tissue with either greater inherent organic stability through decay and diagenesis (e.g., kerogen deriving from plant tissues) or the ability to be converted into a stable inorganic substance (e.g., authigenic pyritization and subsequent oxidation of plant tissues). Thus, we reject the identification of ovarian follicles in STM 10-12 by Bailleul et al.^12^ as insufficiently supported by the evidence available.

### Identification of plant propagules similar to some enantiornithine “follicles” in the botanical record from the Jehol Biota

**Figure 2:**
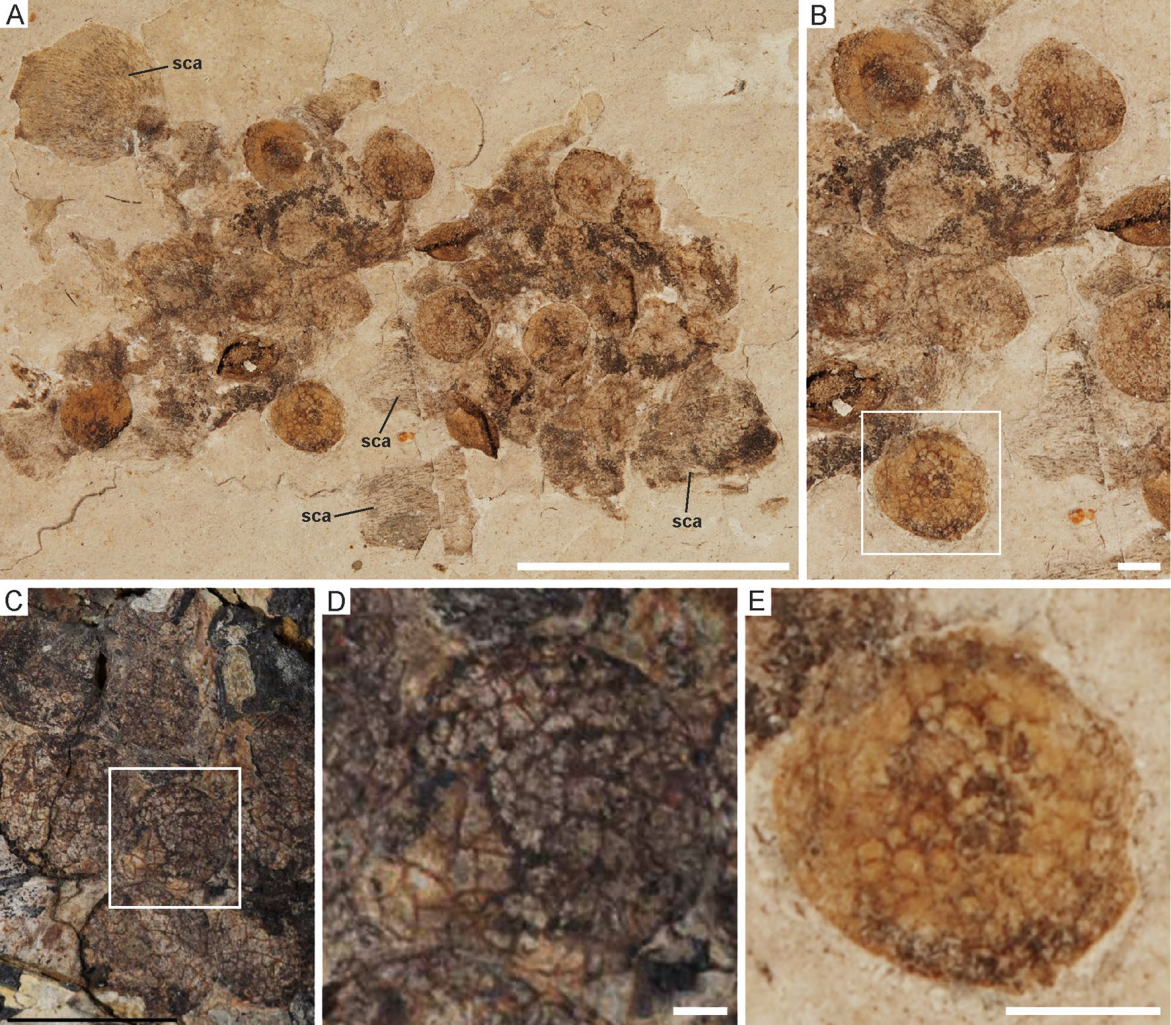
Plant propagules from the botanical record of the Jehol Biota that show a resemblance to some enantiornithine “follicles”. (**A**) Aggregation of *Carpolithes multiseminalis* propagules (NIGPAS PB19001). (**B**) Detail of NIGPAS PB19001 showing propagules with a reticulate surface structure. (**C**) “Follicles” in the enantiornithine fossil STM 29–8 (from Ref. 1: Fig. S5; used with permission). (**D**, **E**) Details of a “follicle” and *C. multiseminalis* propagules from the framed areas in (**B**) and (**C**), respectively (note the different size of the shown structures). Abbreviation: sca, scale or bract. Scale bars equal 10 mm in (**A**) and (**C**) and 1 mm in (**B**, **D**, **E**).

In a recent survey of plant propagules in the fossil record of the Jehol Biota, some isolated aggregations were identified, which show a similar morphology to the “follicles” described for some enantiornithine fossils from the Jehol Biota^38^. These aggregations include propagules, which are assigned to the form taxon *Carpolithes multiseminalis*^39^ and are spherical to slightly ovate, 2.6‒3.8 mm in diameter, with an irregularly reticulate surface with a ‘mesh’ size of ~ 300‒500 µm. Three specimens (NIGPAS PB18986, PB19001 [Fig. 2A, B], PB19004) consist of propagule aggregations preserved with structures resembling striated scales or supporting bracts. These specimens were interpreted as pellets/regurgitates^38^, but are, after careful reconsideration, now considered to more likely be dissociated cones. One further specimen (NIGPAS PB19199), however, shows an aggregation of several propagules that may be interpreted as a pellet/regurgitate or coprolite.

The propagules correspond with the “follicles” of the enantiornithine fossil STM 29-8 in their shape and in that the surface has a reticulate structure (Fig. 2C‒E). With a size of about 2.6‒3.8 mm, the isolated propagules are, however, smaller than those in the enantiornithine specimen STM 29-8, which have a diameter of 5.8‒8.8 mm^1^. The propagules also resemble the “follicles” preserved in the enantiornithine fossil STM 10-12^6^, but, again, the size of the isolated propagules is less than that of the “follicles” in STM 10-12, whose average diameter is 7.7 mm^6^. “Follicles” in the enantiornithine fossil STM 10-45^1^ show an unstructured surface, but resemble crop contents of *Sapeornis* (ref. 40: 164/165), which also suggests that they represent ingested food items.

### LSF imaging of *Eoconfuciusornis zhengi* “follicles”

Ten to twelve “follicles” were counted for the *Eoconfuciusornis zhengi* specimen STM 7-144 (Fig. 1A), which range in size between 3.5‒5.8 mm^8^. It was originally detailed that “[t]he follicles are preserved as slightly three-dimensional voids. The ventral surface of the void bears a circular micro-pattern visible in relief under SEM (…). It is unclear whether the circles are concentric or spiralling (…). This pattern is inferred to be the result of authigenic mineral growth (…), and not a natural morphology of the follicles” (ref. 8: 442). However, we disagree that this pattern relates to authigenic mineral growth rather than original morphology.

Under white light, the follicle-like structures in STM 7-144 appear as circular, grey, shallow (~ 1 mm deep) impressions with a dark black patterning of small, concentric rings around a center dot repeated across the surface. In close association with the “follicles” are several irregularly shaped, paler (somewhat similar to the color of the bones) structures of similar size that are either in flat relief (presumably a split cross section) or preserved as a three-dimensional mass that protrudes above the splitting bed of the slab. Around the “follicles” is a dark black (similar to the color of the feathers) stain in flat relief at the level of the splitting bed.

LSF imaging yields a clearer view of these structures and reveals different intensities of fluorescence. While LSF cannot give precise identification of chemical species, it can show spatial patterns of chemical heterogeneities within a scan to reveal localization patterns. Under LSF, the circular patterning of the “follicles” does not fluoresce, while the rest of the “follicles” appears as yellow-orange-tan. This could indicate a varying chemical composition^41,42^, which is inconsistent with both the interpretation of the ovoid fossils as follicles and the notion that the concentric circles may be the result of authigenic mineral growth. If the structures represent follicles, a homogenous chemical composition and uniform fluorescence emission spectra would be expected, since ovarian follicles do not show a heterogeneous internal structure, and diagenetically induced mineral growth is also unlikely to produce concentric/spiraling circles with different emission spectra.Non-fluorescence, i.e., a black appearance under laser light, is common of carbonaceous matter, because carbon acts to quench fluorescence^41,42^. The blackish circles/spirals are therefore more consistent with an interpretation of the “follicles” as ingested food items. Such is also suggested by the fact that LSF imaging shows a tubercular surface structure of the *Eoconfuciusornis* “follicles” (Fig. 1C). If the organic material was pyritised (or subsequently oxidized into iron oxide) it would also not fluoresce.

The follicle-like structures in *Eoconfuciusornis* were “not considered to be seeds preserved in the stomach on the basis of their morphology and position […], as well as comparison with other types of seeds observed in Jehol fossils” (ref. 8: 507). However, the fossil remains were possibly exposed to some amount of early decay as well as subsequent long-term diagenesis, and it is therefore not expected that stomach contents remained in place if such degradation of the stomach occurred prior to sediment compaction. Irrespective thereof, we note that unambiguous stomach contents from early Cenozoic bird fossils is usually situated in a similar position within the body cavity (Fig. 1B).

## Discussion

To refuse an identification of the “follicles” as stomach contents, it was noted that no seeds with a similar morphology have been identified in the botanical record from the Jehol Biota^1^. Here we rebut this statement by showing that at least the “follicles” in two enantiornithine fossils (STM 29-8 and STM 10-12) closely resemble *Carpolithes multiseminalis* propagules in shape and surface structure. As detailed above, the “follicles” are, however, larger than the *C. multiseminalis* propagules and are therefore likely to stem from different plant species.

Follicles in other enantiornithines have nondescript surface textures that impede a straightforward identification. Overall, however, all “follicles” associated with enantiornithine fossils have similar sizes and shapes (Tab. 1). If a botanical identity can be shown for some of the follicles, it is therefore more reasonable to assume that all of these structures represent ingested food items rather than hypothesizing that some are ovarian follicles and others are stomach contents (it is also most parsimonious based on preservation potential). The mere fact that the “follicles” exhibit different surface textures is more consistent with the assumption that these structures represent ingested food items than with their interpretations as ovarian follicles, which are not expected to show structural variation other than size in different bird taxa.

### Identity of the plant propagules in the enantiornithine fossils

Today, bird-dispersed plant propagules are often, albeit not exclusively^43^, formed by angiosperm plants. However, the Early Cretaceous birds of the Jehol Biota lived in an ecosystem that included only very archaic angiosperms^39,44^ and that was dominated by gymnosperm taxa comprising species of gnetophytes, ginkgoaleans, cycads, bennettites and conifers^38,39,45^.

**Figure 3:**
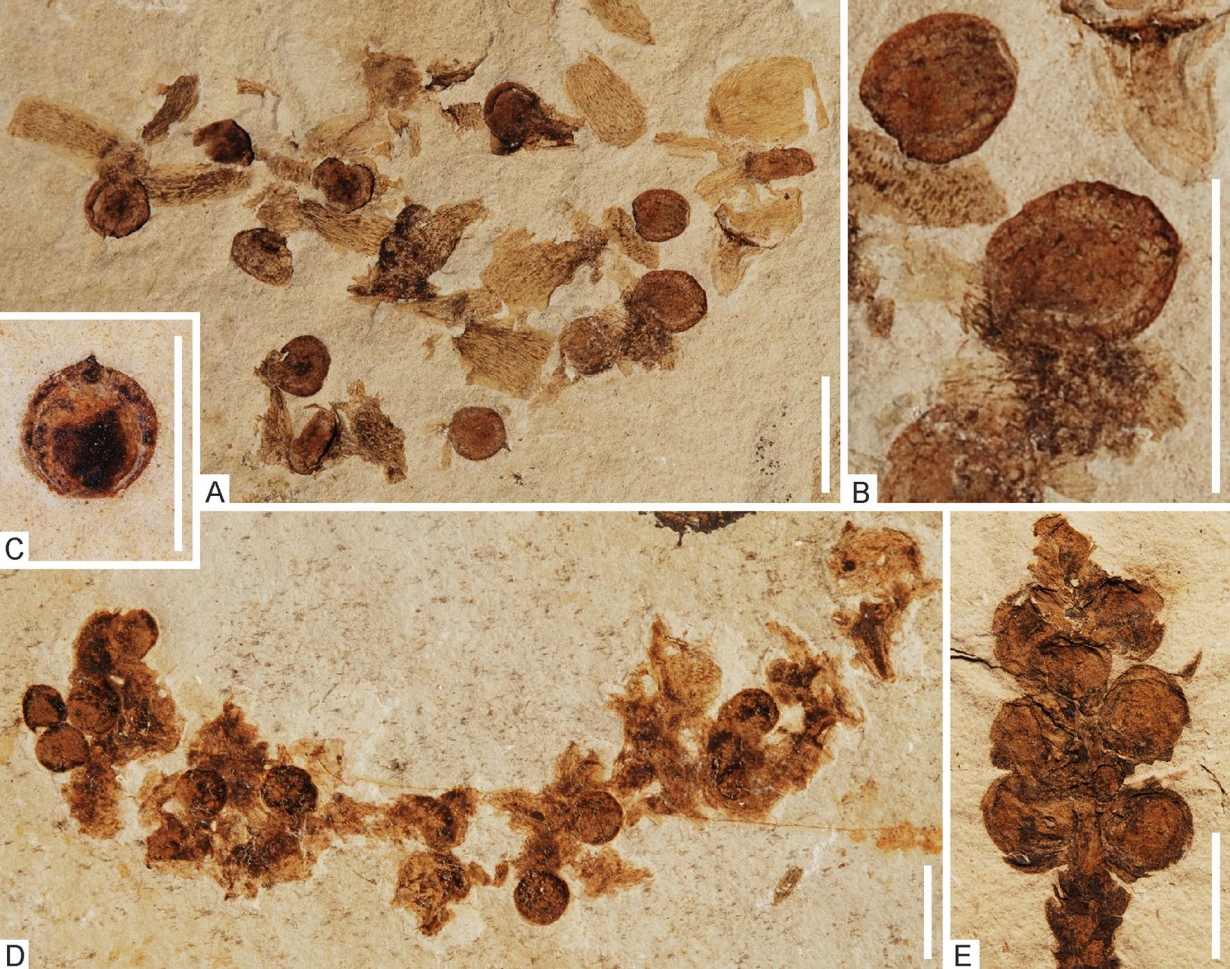
(**A**) Dissociated cone containing *Carpolithes multiseminalis* seeds (NIGPAS PB18986). (**B**) Detail of some seeds with a reticulate surface structure (NIGPAS PB18986). (**C**) Isolated *C. multiseminalis* seed (NIGPAS PB19200). (**D**) Dissociated cone containing *C. multiseminalis* seeds (NIGPAS A20-101). (**E**) *Strobilites taxusoides* cone (NIGPAS PB19149A) with seeds very similar to the *C. multiseminalis* seeds in (**A**–**D**). Scale bars equal 5 mm.

It has been noted that the “follicles” do not conform to any of the known “seed” types from the Jehol Biota^8^ and we could not identify any external structures that would unambiguously support an identification as an ovule or seed (such as a micropyle or a hilum). However, some of the enantiornithine “follicles” (i.e., those preserved in specimens STM 29-8 and STM 10-12) show a close morphological similarity to uncontroversial propagules identified as *Carpolithes multiseminalis* (Fig. 3C). Even though the exact botanical identity of this propagule type is unknown, its association with bracts or scales in specimens NIGPAS PB18986 (Fig. 3A,B), PB19001 (Fig. 2A,B), and PB19004 suggests gymnosperm affinities.

A cone-like organ with propagules very similar if not identical to those of *Carpolithes multiseminalis* was described as *Strobilites taxusoides* (NIGPAS PB19149A [Fig. 3E])^39^. One further cone with a similar arrangement of propagules and bracts/scales is preserved in specimen NIGPAS A20-101/A20-102 (Fig. 3D). For the *S*. *taxusoides* cones a taxodiaceous affinity was assumed, but we note that further unpublished specimens with very similar cones and a distinctive type of sterile foliage preserved in close association, examined by CP at NIGPAS (e.g., NIGPAS PB18324, PB18963, PB19140, PB19154), suggest that the cones most likely were produced by plants that bore sterile foliage allocated to *Liaoningcladus.* This taxon of broad-leaved, apparently deciduous, shoot-dropping conifers with multi-veined leaves has so far been unassigned to any plant group, but is notably common amongst the plant fossils from the Jehol Biota.

Because of their larger size, the “follicles” in the enantiornithine fossils STM 29-8 and STM 10-12 may not represent the same species as the *Carpolithes multiseminalis* propagules from the botanical record of the Jehol Biota, even though we consider it likely that they are from a closely related taxon. The structures being larger in the stomach contents would be consistent with the birds eating only mature propagules, but the uniform sizes of *Carpolithes multiseminalis* seeds in the botanical record argues against the hypothesis that the different sizes represent different growth stages of the propagules. The absence of similar-sized propagules in the botanical record of the Jehol Biota could, however, be explained by the assumption that the birds fed on plant species that did not grow in the lacustrine paleoenvironment in the immediate surroundings of the fossil localities.

### Identity of the *Eoconfuciusornis zhengi* “follicles”

Even though we are confident that the “follicles” in the *Eoconfuciusornis zhengi* fossil likewise represent stomach contents, their true identity remains elusive. The *Eoconfuciusornis* “follicles” clearly differ from *Carpolithes* propagules in their surface structure and have no exact counterpart in the known botanical record from the Jehol Biota. Whereas the “follicles” of the enantiornithine fossils have a reticulate or unstructured surface, those of *E. zhengi* show a tuberculate surface with a circular/spiraling micropattern, which suggests that they derive from a different plant taxon. With regard to their tubercular surface morphology, the *Eoconfuciusornis* “follicles” resemble the ovules of the extant *Afrocarpus* species, such as *A. falcatus* (Podocarpaceae) (Fig. 1D). Admittedly, however, these similarities are very superficial and cannot be taken as evidence for a botanical origin of the *Eoconfuciusornis* “follicles”.

In order to reject a botanical identity of the *Eoconfuciusornis* “follicles”, it was argued that these structures are (1) larger than propagules known to have been ingested by birds, (2) that unambiguously identified plant propagules are only known from crop contents of Mesozoic birds, and (3) that some of the “follicles” are deformed into ovals, which suggests a soft original consistence and conflicts with their identification as plant propagules^8^. Irrespective of the exact nature of the *Eoconfuciusornis* “follicles”, we note that crown group birds ingest considerably larger plant propagules than those found in the fossils from the Jehol Biota^47^ and unambiguous stomach contents consisting of plant propagules are known from *Jeholornis*^48^. This leaves only the argument that compression of the *Eoconfuciusornis* “follicles” indicates a soft consistency. However, soft tissues do not laterally expand in compression fossils^49^, but are two-dimensional projections of three-dimensional tissues.

Another argument against an interpretation of the *Eoconfuciusornis* follicles as stomach contents concerns the observation that “the number of collected confuciusornithiform specimens has been estimated at over 1000, none preserves stomach contents despite the fact that plant propagules are preserved in several specimens of *Jeholornis*, a taxon known from far fewer specimens” (ref. 8: 448). We note, however, that other confuciusornithiforms likewise show no evidence for the preservation of ovarian follicles and most of the fossils belong to the taxon *Confuciusornis*, whereas the fossil record of *Eoconfuciusornis*, which may have differed in feeding ecology, is much sparser and includes only a few published specimens. These observations seem far more consistent with clade-specific dietary tendencies, as opposed to a sample of over 1000 specimens that lacks an example of ovarian follicle preservation.

We furthermore note that plants dependent on or benefitting from animal dispersal commonly produce propagules with a nutritious coat that can attract potential dispersers. Rather than being plant propagules containing the embryo (ovules or seeds), the *Eoconfuciusornis* “follicles” may therefore constitute remnants of fleshy structures that were associated with ovules or seeds. Amongst gymnosperms, a great diversity of such fleshy structures evolved, which attract potential avian dispersers. A fleshy sarcotesta, for example, encloses ginkgo and cycad ovules, and arils (“false fruits”) or equivalent structures (e.g., fleshy epimatia) are formed by a number of taxa, including many Podocarpaceae (Coniferales)^46^. However, the flora of the Jehol Biota also includes gymnosperm taxa with rather unusual reproductive organs, when compared to the modern world, so that an assignment of propagules to particular higher-level taxa usually is not straightforward^38,39^.

### Evolutionary implications

Enantiornithines were often considered to have fed on soft food items^50^, whereas the diet of confuciusornithids has remained elusive^51,52^. Based on cranial morphology and mechanics, the confuciusornithid taxon *Confuciusornis* is regarded to have been a sally-striking predator or a herbivore^53^.

The reinterpreted stomach contents demonstrate that some enantiornithine species ingested plant propagules, but given the relative scarcity of “follicle”-like stomach contents, this should not be taken as evidence that they were habitually frugivorous. The ingestion of plant propagules might seem unexpected in enantiornithines because many species exhibit a toothed beak. However, even some extant squamates with a full dentition act as plant propagule dispersers^54^, and ginkgo ovules, whose original dispersers are unknown, have been regarded as classical “reptile-fruit”^55,56^.

“Follicle”-like stomach contents are known from jeholornithids, confuciusornithids, and enantiornithines. All of these birds are archaic stem group taxa outside Ornithuromorpha, the clade including extant birds. In stark contrast, similar stomach contents have not been reported from Early Cretaceous ornithuromorphs, which are sometimes preserved with large amounts of gastroliths (gizzard stones)^50,57,58^. Gastroliths were also reported from *Jeholornis*^59^ and *Sapeornis*^60^, but are unknown from the numerous specimens of confuciusornithids and enantiornithines found so far^50^, and these differences may indicate disparate feeding ecologies of Early Cretaceous birds.

Gastroliths are often found in birds that feed on coarse plant matter^61^. By adding additional weight load, the high numbers of gastroliths found in the stomach contents of some Early Cretaceous ornithuromorphs are likely to have impaired aerial capabilities and conform to the assumption that these birds predominantly foraged on or near the ground^51,62^. Enantiornithines and confuciusornithids, by contrast, are regarded as predominantly arboreal birds with well-developed perching capabilities^51,62^, and these birds are more likely to have foraged on softer food items that did not require gastroliths to aid in digestion. We believe that a reinterpretation of the putative ovarian “follicles” as stomach contents potentially sheds some new light on the paleobiology of these Mesozoic avian taxa, for which very little direct evidence on feeding habits otherwise exists.

## Methods

Laser-Stimulated Fluorescence imaging was performed with a 405 nm laser following published protocols^41^. 30 s exposures were taken with a Nikon D810 camera and 425 nm blocking filter, and post processing (equalization, saturation and color balance) was performed in Photoshop CS6 software.

The fossils are deposited in the Shandong Tianyu Museum of Nature, Pingyi, China (STM), the paleobotanical collection of the Nanjing Institute of Geology and Palaeontology, Chinese Academy of Sciences, Nanjing, China (NIGPAS), and the Senckenberg Research Institute Frankfurt, Germany (SMF).

## Data Availability

All data and materials related to this paper are available from G.M. (Gerald.Mayr@senckenberg.de).
